# Unilateral Giant Hydronephrosis Secondary to Ureteropelvic Junction Obstruction in a Middle-Aged Woman

**DOI:** 10.1155/2021/9900560

**Published:** 2021-09-03

**Authors:** Masresha S. Dino, Seid M. Hassen, Tesfaye H. Tufa

**Affiliations:** ^1^Department of Surgery, Urology Unit, Addis Ababa, St. Paul's Hospital Millennium Medical College, Ethiopia; ^2^Department of Obstetrics and Gynecology, St. Paul's Hospital Millennium Medical College, Addis Ababa, Ethiopia

## Abstract

**Background:**

Giant hydronephrosis is a rare urologic problem defined as a collection of more than one liter of urine in the collecting system. The radiologic appearance may mimic benign cystic disease of the kidney. We report a case of giant hydronephrosis in a 32-year-old female who presented with progressive abdominal swelling of two-year duration, caused by ureteropelvic junction obstruction with more than nine liters of urine in the collecting system.

**Conclusion:**

Giant hydronephrosis is a rare differential diagnosis for cystic intra-abdominal mass in adults with progressively increasing abdominal swelling. CT and MRI are important in confirming the diagnosis by localizing the origin of the swelling. Management depends on the underlying cause and appearance of the diseased kidney.

## 1. Introduction

Giant hydronephrosis is defined as the presence of more than one-liter urine or 1.6% of the bodyweight of urine in the collecting system [1–3]. The development of hydronephrosis is a gradual process, and most cases are reported to have less than two liters of fluid (urine) collection [3–6]. The common presentation of these patients mimics renal stone disease. Epidemiologically, the left kidney is commonly affected. The most common underlying causes include ureteropelvic junction (UPJ) obstruction and renal stone disease. The majority of this condition will be diagnosed during childhood and infancy (congenital). If not treated early, it may cause progressive and gradual complications like hypertension, kidney rupture, renal failure, and malignant transformation because of prolonged irritation [1–5].

Urolithiasis and tumors either in the renal system or in adjacent organs can cause a compressive disease. This compression leads to obstruction of the renal collecting system leading to hydronephrosis, which gradually grows to have a cystic appearance. There are wide ranges of differential diagnoses for cystic intra-abdominal mass which resemble giant hydronephrosis, including; ovarian cysts, hepatobiliary cysts, renal cell carcinoma (RCC), retroperitoneal tumor, pseudomyxoma, splenomegaly, and ascites [6].

We present a rare case of a 32-year-old woman with long-standing abdominal pain and swelling and who was diagnosed and treated for giant hydronephrosis secondary to UPJ obstruction.

## 2. Case Report

**Figure 1:**
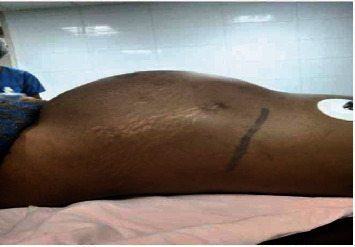
Abdominal examination shows a distended abdomen with cystic appearance on palpation. The marking site is left flank.

A thirty-two-year-old female patient presented with progressive abdominal swelling of two-year duration. The swelling started from the left side of the abdomen and progressed to involve the whole abdomen. The swelling became prominent since one year (Figure 1), with associated dull aching left flank pain. She had no hematuria or any other urinary symptoms. On physical examination, her vital signs were within the normal range with a pulse of 83 beats per minute and blood pressure of 100/80 mmHg. On abdominal examination, she had visible abdominal swelling, which was prominent on the left side of the abdomen. The swelling was cystic with no attachment to the surrounding structure.

**Figure 2:**
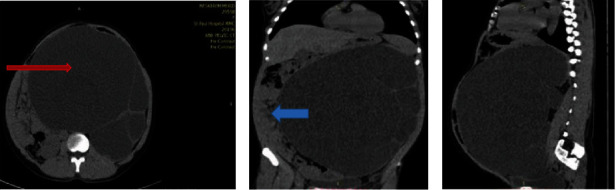
Noncontrast CT scan of the abdomen shows (a) axial view, (b) coronal view, and (c) sagittal view. It shows 20 cm long, 16 cm width cystic swelling (red arrow) with significant mass effect to the surrounding bowel loop (blue arrow).

Laboratory findings of the patient, including urine analysis, serum electrolyte, and hematology profile, were all in the normal range (Table 1). During the initial evaluation, the ultrasound index of the left kidney showed a huge hydronephrosis passing the midline and filling almost the whole abdomen. Computerized tomography (CT) scan of the abdomen without contrast showed massive left kidney pelvicalyceal dilatation with a maximum cortical thickness of 3 mm. The dilatation spans seven vertebral lengths with the inferior border reaching the pelvis (Figure 2). The left ureter and left renal arteries are not visible, and significant mass effects on other abdominal organs were also noted.

**Figure 3:**
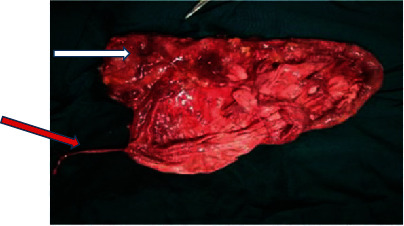
Resected sac (white arrow) with a narrowed left ureter and ureteropelvic junction (red arrow).

A diagnosis of giant left hydronephrosis was made and the patient was prepared for laparotomy. The abdomen was opened with a subcostal flank incision under general anesthesia. Upon entry, 9.5 liters of urine was drained, and a left nephrectomy was performed, which was sent for histopathology. The left ureter was explored and showed severe stenosis at the level of ureteropelvic junction (Figure 3). An intra-abdominal drain was left in situ, and the abdominal wall closed in layers.

**Figure 4:**
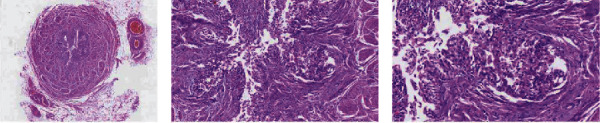
Histopathologic section shows an unremarkable ureteral wall with no increased wall thickness, fibrosis, or inflammation.

She had a smooth postoperative course following the surgery. Her postoperative hematocrit was 21.7%, for which she was transfused with one unit of crossmatched whole blood. Her postoperative serum electrolyte and renal function test were also in the normal range. The intra-abdominal drainage was removed on the 5^th^ postoperative day, and the patient was discharged a day later with satisfactory clinical condition. Histopathologic examination of the biopsy showed a hydronephrotic kidney with no feature of malignant growth (Figure 4).

## 3. Discussion

Giant hydronephrosis with accumulation of more than 9 liters of fluids is an uncommon phenomenon in adults. The first reported case of giant hydronephrosis was in 1746, and etiologies include ureteric obstruction of various causes congenital or acquired, intrinsic or extrinsic, including UPJ obstruction, both renal and ureteric stone disease, and malignancy [1–3]. Patients commonly present with increasing abdominal girth and flank swelling. Other common symptoms include flank pain, hematuria, especially following trauma, and recurrent urinary tract infection. In rare circumstances, they may present with hypertension, obstructive jaundice, intestinal obstruction, respiratory distress, and contralateral hydronephrosis [3]. In our case, the patient presented with progressive abdominal swelling of two years, with associated dull aching sensation and discomfort in the flank area, which is consistent with findings in other literatures.

The first line imaging modality used was ultrasonography as a standard, and the typical finding includes hydronephrosis with a thinned out renal parenchyma. Other imaging modalities were needed to confirm the extension [5, 6]. In addition to accurately diagnosing hydronephrosis, CT and MRI are also used to exclude other causes of intra-abdominal cysts in literature [3]. We had a CT scan done without contrast which confirmed a left hydronephrosis with loss of renal parenchyma. Generally, factors that guide the management of giant hydronephrosis include the following: patient hemodynamic status, presence of functioning kidney, and associated comorbidities such as cardiac illness that can increase surgical morbidity and mortality [1–3]. Children and infants who are diagnosed with these conditions are treated with pyeloplasty which is a rare scenario in adults because of late presentation.

In a patient with a nonfunctioning kidney with loss of renal parenchyma, the recommended treatment is a simple nephrectomy because of the anticipated complication [3]. Percutaneous drainage is an alternative management option in patients with poor clinical parameters. In our case, CT scan revealed a thinned out left renal parenchyma and displaced intra-abdominal organ due to compression by the giant hydronephrosis. Considering the overall clinical picture, performing a nephrectomy was an appropriate decision. Further surgical exploration revealed a narrow left ureter confirming the diagnosis of UPJ obstruction. The patient was followed up for four months postoperatively and had a smooth postoperative course and has resumed her normal activities.

## 4. Conclusion

Giant hydronephrosis secondary to ureteropelvic junction obstruction needs to be considered in adults presenting with huge intra-abdominal cystic mass, especially if they have long-standing pain and progressive abdominal swelling. Imaging studies like CT or MRI are sufficient for diagnosing origin of the mass, but malignancy must be ruled by a histopathologic examination. Treatment of giant hydronephrosis depends on the age, underlying cause, patient clinical condition, and presence or absence of renal parenchyma.

## Figures and Tables

**Table 1:** Important laboratory investigation.

Laboratory tests	Results
Urine analysis	Non revealing
WBC	4200
Hct	41.4%
Creatinine	0.67
Urea	23.3
Potassium	4.53
Sodium	139
Chlorine	101.2

## Data Availability

All data supporting the result are included within the manuscript.
