# Corrigendum to “Unilateral Giant Hydronephrosis Secondary to Ureteropelvic Junction Obstruction in a Middle-Aged Woman”

**DOI:** 10.1155/2022/9813052

**Published:** 2022-03-02

**Authors:** Masresha S. Dino, Seid Mohammed Hassen, Tesfaye H. Tufax

**Affiliations:** ^1^Department of Surgery, Urology Unit, Addis Ababa, St. Paul's Hospital Millennium Medical College, Ethiopia; ^2^Department of Obstetrics and Gynecology, St. Paul's Hospital Millennium Medical College, Addis Ababa, Ethiopia

In the article titled “Unilateral Giant Hydronephrosis Secondary to Ureteropelvic Junction Obstruction in a Middle-Aged Woman” [1], the email address of the co-author Seid M. Hassen needs to be changed. This was a mistake on the authors' behalf, and the correct email address is as follows: from “seid96@yahoo.com” to “seidm96@yahoo.com.”
